# How can we measure psychological safety in mental healthcare staff? Developing questionnaire items using a nominal groups technique

**DOI:** 10.1093/intqhc/mzae086

**Published:** 2024-08-31

**Authors:** Katharina Sophie Vogt, John Baker, Rebecca Coleman, Sarah Kendal, Bethany Griffin, Taha Anjum, Kirsty Louise Ashley, Bethany Lauren Archer, Katherine Berry, Robyn Feldman, Stephanie Gray, Sally Jane Giles, Benjamin James Helliwell, Chelsea Hill, Aimee Elisha Hogan, Magdalena Iwanow, Timon Anton Arie Jansen, Zach Johnson, James A Kelly, Joshua Law, Emily Mizen, Owenvbiugie Omorefe Obasohan, Maria Panagioti, Ffion Smith-Wilkes, Sarah Steeg, Christopher D J Taylor, Natasha Tyler, Sophie Wade, Judith Johnson

**Affiliations:** Bradford Institute for Health Research, Bradford Royal Infirmary, Temple Bank House, Duckworth Lane, Bradford BD9 6RJ, United Kingdom; School of Psychology, University of Leeds, Lifton Place, Leeds LS2 9JT, United Kingdom; Department of Psychology, Institute of Population Health, University of Liverpool, Brownlow Street, Liverpool L69 3GB, United Kingdom; School of Healthcare, University of Leeds, Clarendon Way, Leeds LS2 9JT, United Kingdom; College of Medical, Veterinary and Life Sciences, University of Glasgow, School of Health and Wellbeing, Clarice Pears Building, Glasgow G12 8TB, United Kingdom; School of Healthcare, University of Leeds, Clarendon Way, Leeds LS2 9JT, United Kingdom; Bradford Institute for Health Research, Bradford Royal Infirmary, Temple Bank House, Duckworth Lane, Bradford BD9 6RJ, United Kingdom; School of Psychology, University of Leeds, Lifton Place, Leeds LS2 9JT, United Kingdom; South West Yorkshire Partnership NHS Foundation Trust, Ouchthorpe Lane, Wakefield WF1 3SP, United Kingdom; Independent expert, United Kingdom; Department of Psychology, Institute of Population Health, University of Liverpool, Brownlow Street, Liverpool L69 3GB, United Kingdom; Division of Psychology and Mental Health, University of Manchester, Oxford Road, Manchester, Manchester M13 9PL, United Kingdom; Independent expert, United Kingdom; Independent expert, United Kingdom; NIHR Greater Manchester Patient Safety Research Collaboration, NIHR School for Primary Care Research, Division of Population Health, Health Services Research and Primary Care, University of Manchester, Oxford Road, Manchester M13 9PL, United Kingdom; Pennine Care NHS Foundation Trust, Humphrey House, Angouleme Way, Bury BL9 0EQ, United Kingdom; Independent expert, United Kingdom; Clinical Psychology, Health Innovation Campus, Lancaster University, Sir John Fisher Drive, Bailrigg, Lancaster LA1 4YW, United Kingdom; Secure Forensic Inpatient Service, Lancashire and South Cumbria NHS Foundation Trust, Sceptre Point, Sceptre Way Walton Summit, Preston PR5 6AW, United Kingdom; Mersey Care NHS Foundation Trust, Kings Business Park, Trust Offices/V7 Buildings, Prescot L34 1PJ, United Kingdom; Sheffield Health and Social Care NHS Foundation Trust, Centre Court, Atlas Way, Sheffield S4 7QQ, United Kingdom; Independent expert, United Kingdom; Clinical Psychology, Health Innovation Campus, Lancaster University, Sir John Fisher Drive, Bailrigg, Lancaster LA1 4YW, United Kingdom; Bolton Community Mental Health, Greater Manchester Mental Health NHS Foundation Trust, Bury New Road, Manchester M25 3BL, United Kingdom; Department of Psychology, Institute of Population Health, University of Liverpool, Brownlow Street, Liverpool L69 3GB, United Kingdom; Norwich Medical School, University of East Anglia, Norwich Research Park, Norwich, Norfolk NR4 7TJ, United Kingdom; Tees, Esk and Wear Valley NHS Foundation Trust, West Park Hospital, Edward Pease Way, Darlington, Durham DL2 2TS, United Kingdom; NIHR Greater Manchester Patient Safety Research Collaboration, NIHR School for Primary Care Research, Division of Population Health, Health Services Research and Primary Care, University of Manchester, Oxford Road, Manchester M13 9PL, United Kingdom; Independent expert, United Kingdom; NIHR Greater Manchester Patient Safety Research Collaboration, NIHR School for Primary Care Research, Division of Population Health, Health Services Research and Primary Care, University of Manchester, Oxford Road, Manchester M13 9PL, United Kingdom; Division of Psychology and Mental Health, University of Manchester, Oxford Road, Manchester, Manchester M13 9PL, United Kingdom; Manchester Academic Health Science Centre, University of Manchester, CityLabs 1.0, Nelson Street, Manchester M13 9NQ, United Kingdom; Division of Psychology and Mental Health, University of Manchester, Oxford Road, Manchester, Manchester M13 9PL, United Kingdom; Pennine Care NHS Foundation Trust, Humphrey House, Angouleme Way, Bury BL9 0EQ, United Kingdom; NIHR Greater Manchester Patient Safety Research Collaboration, NIHR School for Primary Care Research, Division of Population Health, Health Services Research and Primary Care, University of Manchester, Oxford Road, Manchester M13 9PL, United Kingdom; Counselling & Mental Health Service, Palatine Centre, Durham University, Stockton Road, Durham DH1 3LE, United Kingdom; Bradford Institute for Health Research, Bradford Royal Infirmary, Temple Bank House, Duckworth Lane, Bradford BD9 6RJ, United Kingdom; School of Psychology, University of Leeds, Lifton Place, Leeds LS2 9JT, United Kingdom; School of Public Health and Community Medicine, University of New South Wales, High Street, Sydney NSW 2052, Australia; Division of Nursing, Midwifery and Social Work, School of Health Sciences, University of Manchester, Jean McFarlane Building, Oxford Road, Manchester M13 9PY, United Kingdom

**Keywords:** mental health, patient safety, psychological safety, healthcare workforce, nominal groups technique

## Abstract

There have been growing concerns about the well-being of staff in inpatient mental health settings, with studies suggesting that they have higher burnout and greater work-related stress levels than staff in other healthcare sectors. When addressing staff well-being, psychological safety can be a useful concept. However, there is no measure of psychological safety that is suitable for use in inpatient mental health settings. Edmondson (1999) is the most commonly used measure of psychological safety, but it was designed for use in general physical healthcare settings. As inpatient mental health settings are unique environments, transferability of knowledge from physical to mental healthcare settings cannot be assumed. We sought to develop questionnaire items that capture psychological safety among healthcare staff working in acute inpatient mental healthcare settings. We used the nominal group technique, a consensus method involving rounds of discussion, idea generation, and item rating/ranking to identify priorities. Twenty-eight stakeholders participated, including 4 who had lived experience of mental health problems, 11 academics and 18 healthcare professionals (8 participants identified with more than 1 category). The study involved a workshop with three parts: (i) an overview of current research and limitations of the Edmondson (1999) measure as outlined above, (ii) discussion on what items should be retained from the Edmondson (1999) measure, and (iii) discussion on what items should be added to the Edmondson (1999) measure. Twenty-one items were generated and retained to capture psychological safety in inpatient mental health settings. These measure professionals’ sense of being valued by their team and organization, feeling supported at work, feeling physically safe and protected from physical harm, and knowing they can raise concerns about risk and safety. This is the first study to generate questionnaire items suitable for measuring staff psychological safety in mental health settings. These have been generated via a consensus method to ensure stakeholders’ views are reflected. Further research is needed to evaluate factor structure, internal reliability, and convergent validity.

## Introduction

There have been growing concerns about the well-being of staff in inpatient mental health settings, with studies suggesting they have higher burnout and greater work-related stress levels than staff in other healthcare sectors [1]. Poor well-being and quality of life of mental healthcare staff has, in turn, been associated with poorer quality of care and patient safety concerns [1–4].

When addressing staff well-being, psychological safety can be a useful concept. Traditionally, this has been defined as the belief that it is safe to take interpersonal risks without a fear of negative consequences [5]. Employees who feel psychologically safe are able to raise concerns, speak up about bad practice or provide feedback, without worrying about negative consequences [6, 7]. Over the past two decades, quantitative research has suggested that psychological safety helps to generate successful outcomes in healthcare teams and prevents patient safety incidents and errors [8–10].

There has been a comparative lack of research into healthcare services and patient safety within mental healthcare settings [11]. To date, the psychological safety literature is limited to predominantly quantitative research focused on physical healthcare settings, with no studies investigating this topic in acute mental health settings. Edmondson’s measure of psychological safety [5] is the most commonly used scale for capturing psychological safety. However, we are aware of no study using either Edmondson’s measure, nor any other measure of psychological safety, which has been conducted in healthcare staff in acute mental health settings, or adapted for this context. Thus, not only is there currently a lack of research on staff psychological safety in acute mental health settings but there is also no measure that saliently captures the concept.

This lack of literature is concerning. A recent evidence synthesis which contained studies from physical healthcare settings [8] highlighted that low staff psychological safety negatively impacted patient care. Findings from a user-led qualitative study with service-users of mental health services and mental health care staff in the UK also linked the desensitization of mental healthcare staff to poorer patient care, i.e. increased patient safety risks [12]. While desensitization does not directly relate to the concept of psychological safety, it does highlight the importance of staffs’ emotional experiences in contributing to patient care quality. Thus, it is important to consider that ascertaining staff psychological safety levels across acute mental health settings, and navigating areas for improvement, would benefit not only staff but also service users.

Transferability of knowledge from physical to mental healthcare settings cannot be assumed. Inpatient mental healthcare is a unique working environment where staff are at high-risk for exposure to, and experience of, violence, while supporting individuals in crisis who often cannot keep themselves, or others, safe [13–16]. As aforementioned, working in this environment can lead to negative outcomes for staff, including poor well-being, lower health-related quality of life, and compassion fatigue [1].

The need for a specialized measure of psychological safety has also been supported by findings from a recent qualitative study [17] with healthcare staff working in acute mental healthcare settings in the UK. The findings suggested that the traditional conceptualization of psychological safety is insufficient to capture their experience of psychological safety. Study participants conceptualized psychological safety as feeling safe from physical harm, developing meaningful relationships with colleagues and service users, and feeling valued at work. Facilitators of psychological safety included an appropriate staffing ratio and skill mix, being able to form meaningful relationships and having access to support, while barriers were reliance on agency workers, punitive management approaches and the physical risks involved in mental health inpatient services.

As such, a quantitative measure of psychological safety that saliently measures the concept of psychological safety in mental health settings is needed, so that further information on experiences of psychological safety could be collected from a larger, more representative sample. Such a measure could be used to evaluate any potential interventions.

## The current study

The current study sought to develop questionnaire items that capture psychological safety in healthcare staff working in acute inpatient mental healthcare settings. This was achieved by amending and extending the measure of psychological safety [5] via the nominal groups technique, which is a consensus method [18, 19]. The nominal group technique brings together experts and stakeholders to reach consensus on a particular topic, such as priorities for guidelines, or survey item generation [19]. The nominal group technique is an established method for survey item generation in health research [18].

Use of the nominal group technique in the current study focused on two main questions, which were [1]: ‘what items of the Edmondson (1999) measure [5] need to be deleted?’, and [2] ‘what items need to be added?’. This approach aimed to create an adapted measure which captured the psychological safety of healthcare professionals working in inpatient mental healthcare in the UK.

## Methods

### Ethical approval

Ethical approval for this study was obtained from the University of Leeds, School of Psychology Ethics Committee (PSCETHS-660).

### Design

The study design was a co-production event using the nominal groups technique, delivered as an online co-design workshop (4 h), with completion of a follow-up survey 2 weeks post-event.

### Participants and recruitment

The research team identified and emailed potential participants who had been pre-identified as lay or professional experts in the areas of psychological safety or inpatient mental healthcare. All were provided with an information sheet and the opportunity to ask questions. Forty individuals were contacted and 26 agreed to take part (uptake rate: 65%). Participants received a £30 voucher and were given the opportunity to contribute as an author to the manuscript. Together with the two lead researchers, the final number of contributors was 28.

Demographic information was provided by 25 participants (missing *n* = 3). The mean age was 35.04 years (SD = 6.86). Four participants had lived experience of mental health difficulties, 11 were academics, and 18 were healthcare professionals, including mental health nurses, clinical psychologists, psychiatry doctors, and healthcare support workers. Eight participants identified with more than one category. Participants were asked to self-describe their ethnicity; with the majority identifying as White British or White Other (*n* = 20), three as British (*n* = 3), one as Greek (*n* = 1) and one as Black African (*n* = 1).

### Procedure

Participants provided consent to participate via an online form. They were asked to complete a baseline demographics questionnaire, attend a 4-h online workshop (hosted on Zoom) and complete a follow-up questionnaire 2 weeks after the workshop.

#### Online workshop

The three-part online workshop comprised: presentation of current state of research and limitations of the Edmondson (1999) measure, followed by discussion of what items should be retained from the Edmondson (1999) measure, and finally, discussions on what items should be added to the Edmondson (1999) measure.

After Part 1 (the presentation), participants were split into small breakout groups of 5–7 participants for Parts 2 and 3. Parts 2 and 3 followed the nominal group technique format, which consists of: silent contemplation of a question where participants can add comments on an online whiteboard; small group discussion; large group discussion and ranking of suggestions/items [19, 20]. Group allocation was pre-planned to ensure an even mix of experience and roles in all groups. Each breakout group had its own online whiteboard hosted on Padlet [21]. Parts 2 and 3 both started with a question being outlined. There was then a 5-min silent contemplation period where participants could add comments on to the whiteboard. This was followed by small group discussions and further comments on the whiteboard. At the end of each part, the breakout groups all returned to the main room to provide feedback to the main group.

#### Criteria for group consensus

There is a lack of standardized cut-offs for deciding when an item should be included or excluded when using the nominal group technique. The lead authors therefore applied criteria which they considered best fit with the group consensus, after each round of discussion. These criteria were designed to capture the group consensus at each stage, consistent with the goals of co-production [22].

#### Consensus seeking

Within this study, there were three rounds of consensus seeking: In Round 1, items were deleted when a majority view indicated they should be deleted. In Round 2, items were retained when the mean score indicated that overall, the group deemed them to be a high priority. In Round 3, items were removed when no-one in the group deemed them to be a priority. The report from the co-production work was then circulated to all authors; all authors were satisfied with the criteria selected and the items selected, confirming that consensus was reached.

##### Round 1: Which items should be deleted from the Edmondson (1999) measure [5]?

Following the large group discussion of Part 2, each participant completed an online survey [hosted on Qualtrics [23] where they were asked to rate all seven items from Edmondson (1999) measure [5] from 1 (‘Definitely delete’) to 5 (‘Definitely keep’)]. If more than 50% of participants rated items as either ‘Definitely delete’ or ‘Probably delete’, items were deleted.

##### Round 2: Which items should be added to the Edmondson (1999) measure [5]?

Following the large group discussion of Part 3, facilitators compiled all suggestions for new items into a list which was uploaded to Qualtrics [23]. Participants were then asked to rate all items (total *n* = 139) from 1 (‘Low priority for inclusion’) to 7 (‘High priority for inclusion’). Sixteen items (*n* = 16) were rated with a mean of 6 or above and were collated by researchers and used as a basis to create a draft version of the new measure.

##### Round 3: Should any items from the draft questionnaire be removed?

The draft measure, containing 24 items, was sent to participants 2 weeks post-event, for final rankings and to check consensus. Participants ranked all items in priority order (1 = highest priority for inclusion, 24 = lowest priority for inclusion); the lower the mean item score, the more important participants thought the items were for inclusion. If an item was not considered a top-five priority by any participant (i.e. all participants considered it a lower priority item), it was excluded.

## Results

### Round 1: Which items should be deleted from the Edmondson (1999) measure?

Twenty-six participants completed this rating. Only two items were retained. These were item 2 (‘Members of this team are able to bring up problems and tough issues’) and 5 (‘It is difficult to ask other members of this team for help’) (Table 1). Following points raised in the large group discussions, both items were then split and adapted into two items, with one pertaining to management and one pertaining to the immediate team. The item ‘It is difficult to ask other members of this team for help’ was divided and adapted into ‘It is difficult to ask senior management for help’ and ‘It is difficult to ask other members of this team for help’. The item, ‘Members of this team are able to bring up problems and tough issues’, was divided and adapted into ‘Members of this team can bring up problems and tough issues in discussion with other team members’ and ‘Members of this team can bring up problems and tough issues in discussion with senior management’.

**Table 1.** Responses to the online survey asking participants to rate items from the Edmondson measure for retention in the new questionnaire

Item	Delete (*n*) (Responses: Definitely delete, probably delete)	Unsure (Responses: ‘don’t know’)	Retain (*n*) (Responses: Probably Keep & Definitely Keep)
If you make a mistake on this team, it is often held against you. (*n*= 26)	14	0	12
Members of this team are able to bring up problems and tough issues. (*n*= 26)	11	1	14
People on this team sometimes reject others for being different. (*n*= 25, *missing n = 1*)	17	2	6
It is safe to take a risk on this team. (*n*= 26)	18	1	5
It is difficult to ask other members of this team for help. (*n*= 26)	10	4	12
No one on this team would deliberately act in a way that undermines my efforts. (*n* = 26)	14	2	10
Working with members of this team, my unique skills and talents are valued and utilized. (*n* = 26)	16	1	9

### Round 2: Which items should be added to the Edmondson (1999) measure?

Participants made 139 novel suggestions of items to add. Twenty-seven participants rated these items. A total of 16 items had a mean score above 6 (on a scale from 1 to 7; Table 2). The lead researchers then made minor amendments to items for clarity and consistency with points raised in the large group discussions (Table 2). These changes included (i) recognizing the distinction between team and whole-organization dynamics (e.g. splitting the item ‘I feel listened to and valued by management and senior professionals’ into ‘I feel valued by senior management within my organization’ and ‘I feel listened to by senior management within my organization’) and (ii) changing wording into ‘I’-statements throughout (e.g. ‘everyone in this team treats one another with respect’ was changed to ‘I feel respected in my team’). Four items were also excluded as they duplicated another, higher rated item (see Table 2).

**Table 2.** Items rated above 6 in the second round.

Initially rated item	*N*	Min	Max	Mean	Topic category	Item following amendments
I am confident that I will be provided with appropriate support by my organization if a serious incident occurs	25	1	7	6.32	Support	I will be provided with appropriate support by my organization if an incident occurs.
I feel listened to and valued by management and senior professionals	26	1	7	6.27	Valued	I feel valued by senior management within my organization. I feel listened to by senior management within my organization.
My organization does not penalize me for speaking out about concerns.	25	3	7	6.20	Patient safety concerns	No amendments made, retained as is.
I feel listened to and valued by other staff on the ward.	25	1	7	6.16	Valued	I feel listened to by other staff on my team. I feel valued by other staff on my team.
Everyone in this team treats one another with respect.	26	4	7	6.15	Respect	I feel respected in my team.
I feel safe to challenge colleagues when they speak about patients in a disrespectful manner.	26	2	7	6.15	Other	I can challenge colleagues when they are disrespectful towards patients.
I feel confident having open discussions around risk within my team.	25	2	7	6.12	Risk	I can have open discussions around risk within my team.
I feel supported when asking my manager for help.	26	2	7	6.12	Support	I feel that my manager is supportive.
I feel supported by my colleagues.	26	2	7	6.12	Support	I feel that my team is supportive.
People in my team are accepting of others regardless of ethnicity, gender, etc.	26	2	7	6.12	Identity	People in my team are accepting of other team members regardless of ethnicity, gender, sexuality or disability.
I feel confident that I can raise concerns about areas of practice and they will be resolved.	25	1	7	6.08	Patient safety concerns	Assesses the same thing as another higher rated item, thus excluded.
I feel emotionally supported post incident.	25	1	7	6.08	Support	Assesses the same thing as another higher rated item, thus excluded.
If an incident occurs, I feel able to ask for support from my team and wider organization.	25	1	7	6.08	Support	Assesses the same thing as another higher rated item, thus excluded.
There is an appreciation for all cultures and backgrounds and diversity is celebrated.	26	2	7	6.08	Identity	Assesses the same thing as another higher rated item, thus excluded.
I have received adequate training and support to work with the level of risk I face in my work.	26	1	7	6.00	Training	No amendments made, retained as is
When I am at work, I know my physical safety will be prioritized.	26	1	7	6.00	Physical safety	No amendments made, retained as is

Group discussions emphasized the relevance of relationships with service-users and physical safety to psychological safety, but no items pertaining to these scored above 6 on this exercise. To address this, the lead researchers added the highest rated item on physical safety to the draft list (‘My organization/team takes my physical safety seriously’, mean: 5.96).

There was less agreement among participants about the inclusion of items pertaining to relationships with service users. As a result, the lead researchers generated four new items based on comments from the group discussions, which were: ‘If I have a concern about my safety around a service user, I am able to express my concerns to the team’, ‘I am able to get to know service users well’, ‘I can develop meaningful relationships with service users’, and ‘I am able to predict the behaviour of service users on my ward’. Altogether, this round resulted in a total of 20 new items.

### Round 3: Should any items from the draft questionnaire be removed?

Participants were asked to rank the 24 items [retained items (*n* = 4), newly generated items (*n* = 20)] in order of importance from 1 (=most important) to 24 (=least important). Twenty participants completed this. Table 3 shows the items and their ranking, including means, medians, and ranges. The lower the mean, the higher the priority for inclusion.

**Table 3.** Results of the ranking of items

Item ranked	Min	Max	Mean (SD)	Median	Retained or excluded
I will be provided with appropriate support by my organization if an incident occurs.	1	22	6.40 (5.88)	5.00	Retained
I feel valued by senior management within my organization.	1	18	9.10 (5.11)	7.50	Retained
I feel listened to by senior management within my organization.	2	22	9.40 (5.43)	8.00	Retained
My organization does not penalize me for speaking out about concerns.	1	22	8.50 (4.85)	8.50	Retained
I feel valued by other staff on my team.	1	21	8.85 (6.37)	8.50	Retained
I have received adequate training and support to work with the level of risk I face in my work.	1	20	8.95 (6.14)	8.50	Retained
I feel listened to by other staff on my team.	2	21	9.20 (4.53)	9.50	Retained
I feel that my manager is supportive.	2	21	10.15 (5.99)	9.50	Retained
I feel that my team is supportive.	1	22	9.60 (6.84)	9.50	Retained
I feel respected in my team.	1	17	9.20 (4.83)	10.00	Retained
I can challenge colleagues when they are disrespectful towards patients.	3	24	11.75 (6.89)	10.00	Retained
I can have open discussions around risk within my team.	2	21	11.35 (5.46)	11.50	Retained
People in my team are accepting of other team members regardless of ethnicity, gender, sexuality, or disability.	2	24	11.60 (6.18)	11.50	Retained
When I am at work, I know my physical safety will be prioritized.	1	23	11.10 (7.39)	12.00	Retained
If I have a concern about my safety around a service user, I am able to express my concerns to the team.	1	20	12.45 (5.45)	12.50	Retained
My organization/team takes my physical safety seriously.	1	23	12.55 (6.94)	14.50	Retained
Members of this team can bring up problems and tough issues in discussion with other team members.	4	23	15.05 (6.35)	16.50	Retained
Members of this team can bring up problems and tough issues in discussion with senior management.	5	24	15.65 (5.85)	16.50	Retained
I can develop meaningful relationships with service users.	2	23	15.40 (6.41)	18.00	Retained
I am able to get to know service users well.	10	24	18.55 (3.66)	19.00	Excluded
I am able to predict the behaviour of service users on my ward.	9	24	20.15 (4.06)	20.00	Excluded
There is an appreciation for all cultures and backgrounds in my organization.	1	24	17.85 (5.54)	20.00	Retained
It is difficult to ask other members of this team for help.	3	23	17.95 (7.03)	21.50	Retained
It is difficult to ask senior management for help.	6	24	19.25 (5.88)	21.50	Excluded

There was a range of ranks given to each item (range of median scores = 5.00–21.50). Only three items were not ranked as a top-5 priority for inclusion by any participant: ‘I am able to get to know service users well’ [range 10–23, mean: 18.55 (SD 3.66), median: 19.00], ‘It is difficult to ask senior management for help’ [range: 6–24, mean: 19.25 (SD: 5.88), median: 21.50], and ‘I am able to predict the behaviour of service users on my ward’ [range: 9–24, mean: 18.55 (SD: 4.06), median: 19.00]. Two of these three items were added by (KV) and (JJ) after Part 2, and the third item was derived from the Edmondson measure [5]. As a result, these items were removed from the draft questionnaire. The final version contained three items which were adapted from the Edmonson measure, these were ‘Members of this team can bring up problems and tough issues in discussion with other team members’, ‘Members of this team can bring up problems and tough issues in discussion with senior management’, and ‘It is difficult to ask other members of this team for help’.

## Discussion

The aim of this study was to develop questionnaire items to measure psychological safety for healthcare staff working in inpatient mental healthcare wards, by extending and amending the Edmondson measure of psychological safety [5], via the nominal group technique.

### Statement of principal findings

The current study extends the literature in two main ways. Firstly, this is the first study to investigate the concept of psychological safety in the context of healthcare staff working in acute inpatient mental health settings. No published studies have investigated this concept in this group. Participants in the current study were able to relate to the concept outlined by Edmondson, which defines psychological safety as interpersonal risk-taking. Consistent with Edmondson’s conceptualization, participants identified being able to talk about tough issues with colleagues and being able to ask for help as being key aspects of psychological safety for staff working in inpatient mental health services. This is also evident by the retention of two items of the Edmondson measure, suggesting the items were perceived as relevant and salient to the inpatient mental healthcare setting.

However, the group discussion, alongside recent qualitative findings [17] suggested that Edmondson’s concept misses crucial aspects of psychological safety of healthcare staff in the context of inpatient mental health services. Inpatient mental healthcare services are unique work environments, in which service users are often treated when they pose a risk to their own, or others, safety [24]. This explains why item 4 (‘It is safe to take a risk on this team’), for example, was not retained.

### Interpretation within the context of the wider literature

The word ‘risk’ in the context of the inpatient mental healthcare setting has a different connotation from ‘risk’ in other healthcare settings; indeed, there is a considerable literature on therapeutic risk-taking and risk aversion in inpatient mental healthcare settings [25]. This is a key difference between the traditional definition of psychological safety, and psychological safety for the context of inpatient mental healthcare staff. Interestingly, participants suggested adding the item ‘My organization does not penalise me for speaking out about concerns’. Indeed, raising concerns can be interpreted as risk-taking behaviour, as it is well known that speaking-up behaviours, such as whistleblowing, can be associated with negative outcomes [26]. This thus suggests that, while different language is needed, feeling able to take risks does also relate to psychological safety in inpatient mental healthcare, but that the word ‘risk’ is inappropriate for the context. Another item, which was added by participants, was ‘I can have open discussions around risk within my team’. While this item does contain the word risk, it relates to feeling able to raise concerns within the team about feeling at risk around a service user or situation, and being able to speak openly about this, rather than take a risk per se. As such, the current findings suggested risk-taking is still relevant in mental health inpatient services, how it is defined and presented needs careful consideration when compared with general healthcare settings.

The second way in which this study extends the literature is by proposing questionnaire items measuring psychological safety in healthcare staff working in inpatient mental healthcare, as co-produced by experts and stakeholders in the field of inpatient mental healthcare. To date, the Edmondson measure of psychological safety [5] is the most used measure of psychological safety [8]. Other measures of psychological safety that have been used across the literature include Liang *et al*. (2012)’s 5-item measure [27], Detert *et al*. (2007)’s 3-item scale [28], the psychological safety subscales of the Speaking up about Patient Safety Psychological safety questionnaire [7], and the measure of organizational culture in cardiology [29]. However, no existing measure of psychological safety captures the experiences of staff working in inpatient mental healthcare.

It is important to acknowledge that psychological safety is influenced by cultural norms and expectations. For example, in one study of three international teams from one engineering organization, it was found that the concept of psychological safety was similar in the teams from the USA and France, but it differed in the team from Thailand [30]. Furthermore, a study in students explored the relative benefits of teaching approaches which recognized and valued cultural differences, to those which attempted to be ‘blind’ to these differences [31]. Results suggested that approaches which ignored cultural differences were associated with poorer psychological safety in students, highlighting the importance of recognizing and appreciating cultural variations when establishing psychological safety [31]. We recognize that the measure we have created is similarly likely to have been influenced by UK cultural norms and values. However, as ours is the first study anywhere globally to develop a measure of psychological safety for mental healthcare settings, we consider it a starting point for further exploration in other countries, and anticipate adapted versions could be needed depending on the cultural norms and values of those countries.

### Implications for policy, practice, and research

Further validation studies (including factor analysis, re-test reliability, internal reliability and tests of convergent validity with other questionnaires, including the Edmonson questionnaire) will be required before the questionnaire can be finalized. The current study can be considered a key first step to providing a suitable, tailored measure of psychological safety for healthcare workers in UK inpatient mental health settings. Similarly, a validated version of the measure could also be used to understand how psychological safety relates to staff well-being, burnout, and patient safety, advancing the research area with healthcare staff in acute mental health settings.

Current research has identified that there is low psychological safety for staff working in inpatient mental healthcare settings in the UK [17], and that interventions to increase psychological safety are urgently needed. The current measure could be used as a tool to measure the efficacy of interventions and to understand differences between organizations. Similarly, there is scope for international collaboration to explore the concept of psychological safety and applicability of the measure in diverse mental health settings and in different countries across the world. This could be valuable, as studies indicate that burnout levels vary internationally, with the lowest levels in European and central Asian countries, and the highest levels in Sub-Saharan Africa [32]. This could be explored through utilizing the nominal groups technique, or alternative consensus methods (i.e. a Delphi study), to assess the acceptability and cross-cultural validity of the candidate items developed in the current study. Similar to the adaptation approach we have used here, it is likely that some items may need to be removed when culturally adapting this measure, some may need amending and some may need adding. We recognize that this process may also be more complex than the adaptation reported in the current study, due to variations in language and the concepts contained within the measure, which may not translate. Equally, other cultures and languages may hold important concepts in relation to psychological safety which cannot be easily understood from a western, English-speaking mindset. We believe the use of consensus methods will enable these differences to be identified and managed.

### Strengths and limitations

One of the disadvantages of the nominal groups technique is that the perspective of included participants can bias item-generation/selection [18]. While the study used only UK-based participants, it did include the perspectives of different healthcare professionals, service users, and researchers, as well as a chaplain at a mental health hospital; consequently, ensuring a diverse sample representing different perspective and views. Nonetheless, our study was limited by the nature of the participant sample, and to ensure applicability to different populations outside of the UK, further validation with a bigger sample, as well as with participants from different countries and cultural backgrounds is essential.

The study attempted to include non-white participants, yet the researchers acknowledge that there is an under-representation of non-white participants, which may have impacted on results. The proposed survey items should be considered a starting point for further research which will undertake psychometric assessment and validation. It is imperative that the validation process and future studies utilizing this measure include a more diverse sample to build on and adapt the current work.

## Conclusions

This study provides the first draft measure of psychological safety for healthcare staff working in inpatient mental health settings. It is based on the collective experience and expertise of 28 stakeholders and experts in the field of mental healthcare, patient safety, and clinical psychology. Future research must assess the psychometric properties of the measure, to provide insight factor structure, and ensure assessment of validity and reliability.

## Data Availability

List of items and scores may be requested via email from Dr KS Vogt, after publication of results.

## References

[1] Johnson J, Hall LH, Berzins K et al. Mental healthcare staff well-being and burnout: a narrative review of trends, causes, implications, and recommendations for future interventions. *Int J Ment Health Nurs* 2018;27:20–32. doi: 10.1111/inm.12416 29243348

[2] Garcia CL, Abreu LC, Ramos JLS et al. Influence of burnout on patient safety: systematic review and meta-analysis. *Medicina (Kaunas)* 2019;55:553. doi: 10.3390/medicina55090553 PMC6780563 31480365

[3] Hall LH, Johnson J, Watt I et al. Healthcare staff wellbeing, burnout, and patient safety: a systematic review. *PLoS One* 2016;11:e0159015. doi: 10.1371/journal.pone.0159015 PMC4938539 27391946

[4] Keers RN, Plácido M, Bennett K et al. What causes medication administration errors in a mental health hospital? A qualitative study with nursing staff. *PLoS One* 2018;13:e0206233. doi: 10.1371/journal.pone.0206233 PMC6203370 30365509

[5] Edmondson A . Psychological safety and learning behavior in work teams. *Adm Sci Q* 1999;44:350–83. doi: 10.2307/2666999

[6] Okuyama A, Wagner C, Bijnen B. Speaking up for patient safety by hospital-based health care professionals: a literature review. *BMC Health Serv Res* 2014;14:61. doi: 10.1186/1472-6963-14-61 PMC4016383 24507747

[7] Richard A, Pfeiffer Y, Schwappach DDL. Development and psychometric evaluation of the speaking up about patient safety questionnaire. *J Patient Saf* 2021;17:e599–e606. doi: 10.1097/PTS.0000000000000415 28858000

[8] Grailey KE, Murray E, Reader T et al. The presence and potential impact of psychological safety in the healthcare setting: an evidence synthesis. *BMC Health Serv Res* 2021;21:773. doi: 10.1186/s12913-021-06740-6 PMC8344175 34353319

[9] Hunt DF, Bailey J, Lennox BR et al. Enhancing psychological safety in mental health services. *Int J Ment Health Syst* 2021;15:33. doi: 10.1186/s13033-021-00439-1 PMC8045992 33853658

[10] Swendiman RA, Edmondson AC, Mahmoud NN. Burnout in surgery viewed through the lens of psychological safety. *Ann Surg* 2019;269:234–5. doi: 10.1097/SLA.0000000000003019 30169397

[11] Kilbourne AM, Keyser D, Pincus HA. Challenges and opportunities in measuring the quality of mental health care. *Can J Psychiatry* 2010;55:549–57. doi: 10.1177/070674371005500903 20840802 PMC3084488

[12] Carr S, Hafford-Letchfield T, Faulkner A et al. “Keeping Control”: a user-led exploratory study of mental health service user experiences of targeted violence and abuse in the context of adult safeguarding in England. *Health Soc Care Community* 2019;27:e781–e92. doi: 10.1111/hsc.12806 31257700 PMC6852426

[13] Bekelepi N, Martin P. Experience of violence, coping and support for nurses working in acute psychiatric wards. *S Afr J Psychiatr* 2022;28:1700. doi: 10.4102/sajpsychiatry.v28i0.1700 PMC9210159 35747336

[14] d’Ettorre G, Pellicani V. Workplace violence toward mental healthcare workers employed in psychiatric wards. *Saf Health Work* 2017;8:337–42. doi: 10.1016/j.shaw.2017.01.004 29276631 PMC5715456

[15] Renwick L, Lavelle M, James K et al. The physical and mental health of acute psychiatric ward staff, and its relationship to experience of physical violence. *Int J Ment Health Nurs* 2019;28:268–77. doi: 10.1111/inm.12530 30152005

[16] Weltens I, Bak M, Verhagen S et al. Aggression on the psychiatric ward: prevalence and risk factors. A systematic review of the literature. *PLoS One* 2021;16:e0258346. doi: 10.1371/journal.pone.0258346 PMC8500453 34624057

[17] Vogt KS, Baker J, Morys-Edge M et al. I think the first priority is physically safe first, before you can actually get psychologically safe: staff perspectives on psychological safety in inpatient mental health settings. *J Psychiatr Ment Health Nurs* 2024. doi: 10.1111/jpm.13101 PMC11891413 39283719

[18] Harb SI, Tao L, Peláez S et al. Methodological options of the nominal group technique for survey item elicitation in health research: a scoping review. *J Clin Epidemiol* 2021;139:140–8. doi: 10.1016/j.jclinepi.2021.08.008 34400255

[19] McMillan SS, King M, Tully MP. How to use the nominal group and Delphi techniques. *Int J Clin Pharm* 2016;38:655–62. doi: 10.1007/s11096-016-0257-x 26846316 PMC4909789

[20] Varga-Atkins T, Bunyan N, Fewtrell R. The Nominal Group Technique - a practical guide for facilitators. Written for the ELESIG SMall Grants Scheme. Version 1.02011 October 2011. https://www.liverpool.ac.uk/media/livacuk/cll/eddev-files/iteach/pdf/guide_for_ELESIG_v1.pdf (5 May 2023, date last accessed).

[21] Padlet . About us: Meet the Padlet Squad online: padlet.com. 2023. https://padlet.com/about/about-us-3n0xnxd514ce (6 April 2023, date last accessed).

[22] Bandola-Gill J, Arthur M, Leng RI. What is co-production? Conceptualising and understanding co-production of knowledge and policy across different theoretical perspectives. *Evid Policy* 2023;19:275–98. doi: 10.1332/174426421X16420955772641

[23] Qualtrics . 2023. https://www.qualtrics.com/uk/?rid=ip&prevsite=en&newsite=uk&geo=GB&geomatch=uk (16 September 2023, date last accessed).

[24] Bowers L, Chaplin R, Quirk A et al. A conceptual model of the aims and functions of acute inpatient psychiatry. *J Ment Health* 2009;18:316–25. doi: 10.1080/09638230802053359

[25] Manuel J, Crowe M. Clinical responsibility, accountability, and risk aversion in mental health nursing: a descriptive, qualitative study. *Int J Ment Health Nurs* 2014;23:336–43. doi: 10.1111/inm.12063 24548741

[26] Lim CR, Zhang MWB, Hussain SF et al. The consequences of whistle-blowing: an integrative review. *J Patient Saf* 2021;17:e497–e502. doi: 10.1097/PTS.0000000000000396 28671913

[27] Liang J, Farh CIC, Farh J-L. Psychological antecedents of promotive and prohibitive voice: a two-wave examination. *Acad Manage J* 2012;55:71–92. doi: 10.5465/amj.2010.0176

[28] Detert J, Burris E. Leadership behavior and employee voice: is the door really open? *Acad Manage J* 2007;50:869–84. doi: 10.5465/amj.2007.26279183

[29] Bradley EH, Brewster AL, Fosburgh H et al. Development and psychometric properties of a scale to measure hospital organizational culture for cardiovascular care. *Circ Cardiovasc Qual Outcomes* 2017;10:e003422. doi: 10.1161/CIRCOUTCOMES.116.003422 28302647

[30] Cauwelier P, Ribière VM, Bennet A. Team psychological safety and team learning: a cultural perspective. *Learn Organ* 2016;23:458–68. doi: 10.1108/TLO-05-2016-0029

[31] De Leersnyder J, Gündemir S, Ağirdağ O. Diversity approaches matter in international classrooms: how a multicultural approach buffers against cultural misunderstandings and encourages inclusion and psychological safety. *Stud Higher Educ* 2022;47:1903–20. doi: 10.1080/03075079.2021.1983534

[32] Woo T, Ho R, Tang A et al. Global prevalence of burnout symptoms among nurses: a systematic review and meta-analysis. *J Psychiatr Res* 2020;123:9–20. doi: 10.1016/j.jpsychires.2019.12.015 32007680

